# Neural oscillatory deficits in schizophrenia predict behavioral and neurocognitive impairments

**DOI:** 10.3389/fnhum.2015.00371

**Published:** 2015-07-01

**Authors:** Antígona Martínez, Pablo A. Gaspar, Steven A. Hillyard, Stephan Bickel, Peter Lakatos, Elisa C. Dias, Daniel C. Javitt

**Affiliations:** ^1^Nathan Kline Institute for Psychiatric ResearchOrangeburg, NY, USA; ^2^Department of Neurosciences, University of California, San DiegoLa Jolla, CA, USA; ^3^Department of Psychiatry, School of Medicine, ICBM, University of ChileSantiago, Chile; ^4^Department of Neurology, Albert Einstein College of MedicineBronx, NY, USA; ^5^Columbia University, College of Physician and SurgeonsNew York, NY, USA

**Keywords:** schizophrenia, oscillations, attention, magnocellular, ERP, alpha, ERD

## Abstract

Paying attention to visual stimuli is typically accompanied by event-related desynchronizations (ERD) of ongoing alpha (7–14 Hz) activity in visual cortex. The present study used time-frequency based analyses to investigate the role of impaired alpha ERD in visual processing deficits in schizophrenia (Sz). Subjects viewed sinusoidal gratings of high (HSF) and low (LSF) spatial frequency (SF) designed to test functioning of the parvo- vs. magnocellular pathways, respectively. Patients with Sz and healthy controls paid attention selectively to either the LSF or HSF gratings which were presented in random order. Event-related brain potentials (ERPs) were recorded to all stimuli. As in our previous study, it was found that Sz patients were selectively impaired at detecting LSF target stimuli and that ERP amplitudes to LSF stimuli were diminished, both for the early sensory-evoked components and for the attend minus unattend difference component (the Selection Negativity), which is generally regarded as a specific index of feature-selective attention. In the time-frequency domain, the differential ERP deficits to LSF stimuli were echoed in a virtually absent theta-band phase locked response to both unattended and attended LSF stimuli (along with relatively intact theta-band activity for HSF stimuli). In contrast to the theta-band evoked responses which were tightly stimulus locked, stimulus-induced desynchronizations of ongoing alpha activity were not tightly stimulus locked and were apparent only in induced power analyses. Sz patients were significantly impaired in the attention-related modulation of ongoing alpha activity for both HSF and LSF stimuli. These deficits correlated with patients’ behavioral deficits in visual information processing as well as with visually based neurocognitive deficits. These findings suggest an additional, pathway-independent, mechanism by which deficits in early visual processing contribute to overall cognitive impairment in Sz.

## Introduction

Schizophrenia (Sz) is a severe neuropsychiatric disorder associated with prominent cognitive impairment (Kahn and Keefe, 2013). Deficits have been studied extensively in regard to higher level processes such as attention, working memory and executive processing (Nuechterlein et al., 2008). In recent years, however, sensory processing deficits have also been identified using neurophysiological approaches and progress has been made identifying their underlying mechanisms and their contribution to neuropsychiatric and behavioral impairments in Sz (reviewed in Javitt, 2009).

In the visual system, low level processing deficits have been shown to contribute to higher order impairments in Sz patients, including face emotion processing (Butler et al., 2009; Bedwell et al., 2013), reading (Martínez et al., 2013; Revheim et al., 2014), object recognition (Sehatpour et al., 2010), contextual encoding (Dias et al., 2011). Additionally, recent studies have shown that visual and auditory sensory processing deficits converge to impair reading abilities, which may be an early marker of Sz and a key contributor to poor psychosocial outcome (Martínez et al., 2013; Revheim et al., 2014).

Visual processing deficits in Sz patients are most severe in response to stimuli that preferentially activate the magnocellular/dorsal pathway (e.g., stimuli of low contrast and low spatial frequencies; Javitt, 2009; González-Hernández et al., 2014; #3245). For example, we recently found that Sz patients exhibited selective deficits in the generation of sensory-evoked event related potentials (ERPs) in response to stimuli of low spatial frequency (LSF) but not high spatial frequency (HSF). Patients also showed pronounced deficits in feature-selective attention to LSF but not HSF stimuli, as reflected in the amplitude of the selection negativity (SN) component of the ERP (Martínez et al., 2012). These ERP results were paralleled by reduced functional MRI activation and differential behavioral deficits (e.g., reduced hit rate) for LSF compared to HSF stimuli, consistent with a selective magnocellular pathway dysfunction (see also Butler et al., 2001, 2007; Martínez et al., 2008). While these findings suggest that the observed deficits reflect differential impairment of the magnocellular pathway, they may not account for all sensory processing deficits in Sz. In particular, significant, albeit less severe, deficits have also been observed for HSF stimuli (Slaghuis, 1998), suggesting the existence of additional mechanisms of visual information processing impairments in Sz that affect both the magnocellular and parvocellular pathways.

Selective attention in the visual system is typically associated with event-related desynchronization (ERD) of the ongoing alpha-band (7–14 Hz) activity (reviewed in Klimesch, 2012). It has been proposed that alpha activity reflects active inhibition of visual cortical regions during periods when visual processing is not required while the blocking, or desynchronization, of alpha occurs when these regions are brought “on-line” for processing visual information in response to task demands (reviewed in Foxe and Snyder, 2011). Although deficits in alpha generation (Lemere, 1936; Liberson, 1945; Blum, 1957; Boutros et al., 2008) and alpha-blocking (MacMahon and Walter, 1938) were first reported in Sz in the 1930’s, the tools for detailed analysis in the frequency domain have not been readily available until recently.

Here, we utilize a time-frequency approach to measure phase synchronization and single trial modulations of ongoing oscillatory activity during a feature-based selective attention paradigm. The aim was to investigate the possibility that impaired spectral oscillations may contribute to behavioral and neurocognitive deficits in Sz. Specifically, we investigated whether impaired phase synchrony and stimulus-driven alpha ERD may further contribute to deficits in visual information processing and attention in Sz (over and above the well-documented magnocellular pathway processing deficits) and, furthermore, whether these deficits are associated with neurocognitive measures of behavior and performance.

Although oscillations in the frequency domain, in particular within the alpha band, have been extensively characterized in spatial-attention paradigms (Worden et al., 2000; Foxe and Snyder, 2011; Mishra et al., 2012), this is the first study of which we are aware to investigate the effects of feature-selective attention on alpha modulation either in healthy volunteers or Sz patients.

## Materials and Methods

### Subjects

Informed consent was obtained from all subjects following full explanation of the procedures. All participants were compensated monetarily for their participation. Subjects were 19 patients (17 male, mean age 36.5 ± 15.7 years S.D.) meeting DSM-IV criteria for Sz as assessed by Structured Clinical Interview for DSM-IV (SCID; First et al., 1997) and 19 healthy volunteers (15 male, mean age 37.7 ± 15.4 years S.D.) with no history of SCID-defined axis I psychiatric disorders. Sz patients were recruited from stable inpatient and residential care facilities in Rockland County, NY, and all were on a stable dose of antipsychotics (729 ± 529 S.D. chlorpromazine equivalents/day) at the time of testing. Patients and control volunteers were excluded if they had any neurological or ophthalmologic disorders that might affect performance, or if they met criteria for alcohol or substance dependence within the last 6 months or alcohol/substance abuse within the last month. All participants had visual acuity corrected to at least 20/32 (0.63) on the Logarithmic Visual Acuity Chart (Precision Vision). Informed consent was obtained from all subjects following full explanation of all procedures.

Subject groups did not differ significantly in age (*t*_(36)_ = 0.58, *p* = 0.56) or Edinburgh score for handedness (*t*_(36)_ = −1.51, *p* = 0.14). Compared to control subjects, Sz patients had fewer years of formal education (*t*_(36)_ = −5.06, *p* < 0.001) along with lower Quick IQ scores (Ammons and Ammons, 1962; *t*_(36)_ = 3.04, *p* = 0.004) and reduced Hollingshead socioeconomic status (SES) scores (*t*_(36)_ = 7.77, *p* < 0.001). Parental SES, however, did not differ significantly between the groups (*t*_(36)_ = 1.86, *p* = 0.07), suggesting relatively similar premorbid backgrounds (Table 1).

**Table 1:** **Subject demographics**.

Characteristic	Controls (*n* = 17)	Patients (*n* = 19)
Age, years	38	37
Sex (male/female)	15/2	17/2
CPZ Equivalents, mg		729.7 (97.2)
Anti-Psychotics, *n*		
Typical		2
Atypical		15
Combination		2
IQ (Quick Test)	104 (3.5)	92.8 (1.9)
Years of Education	14.5 (0.5)	11.5 (0.4)
Illness Duration, years		14.1 (2.2)
Participant SES	45.4 (2.4)	23.14 (1.5)
Parental SES	44.3 (3.1)	35.6 (2.4)

*Abbreviations: CPZ, Chlorpromazine; IQ, Intelligence Quotient, (Ammons and Ammons, 1962); SES, socioeconomic status, measured by 4-factor Hollingshead Scale (Hollingshead, 1975). Standard errors are given in parentheses*.

### Stimuli and Task

**Figure 1:**
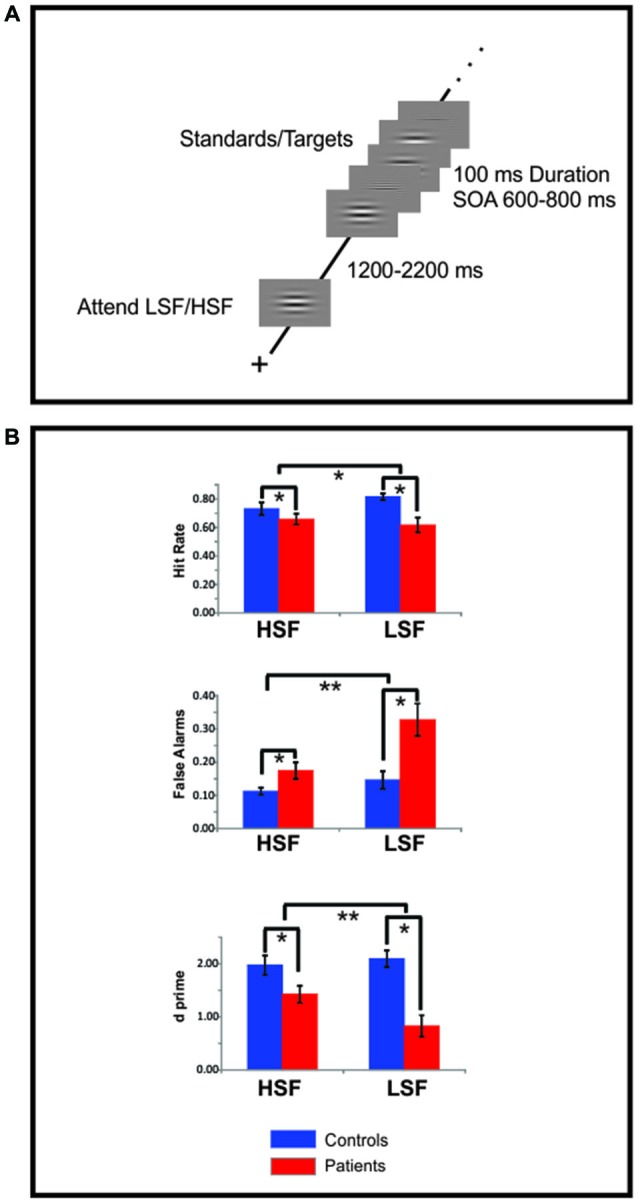
**Experimental design and behavioral data. (A)** Each block of trials began with a 1000 ms presentation of either a high spatial frequency (HSF) or low spatial frequency (LSF) sinusoidal gabor pattern indicating the to-be-attended spatial frequency (SF). After a variable delay of 1200–2200 ms a random sequence of high and low SF patterns was presented, the subjects’ task was to selectively attend to the cued SF and respond to infrequent targets in that SF channel. **(B)** Hit rate (top row) false alarms (FAs) rate (middle row) and d-prime measures (bottom row) of target detection performance for high (HSF) and low SF (LSF) targets for Sz patients (red bars) and control subjects (blue bars). Compared to control subjects, patients with Sz were deficient on all measures (statistical significance is indicated by asterisks). For all these measures Sz performance was more impaired for low, compared to high, SF targets. Error bars are standard error of the mean.

All stimuli were delivered with Presentation software^1^ on a 19” ViewSonic LCD monitor situated 100 cm in front of the subject. Subjects were seated in a comfortable chair within an electrically shielded, sound-attenuated, dimly lit, experimental chamber. Stimuli were horizontal gratings sinusoidally modulated with a 2D Gaussian envelope to form a circle; the grating frequency was either 0.8 cycles per degree (cpd, LSF standards or 5 cpd HSF standards; Figure 1A). The circular gratings subtended 6.5° of visual angle from the center to their outer edge and were delivered against a gray field that was isoluminant with the mean luminance of the gratings. Target stimuli consisted of infrequent (20%) gratings having a slightly higher (6 cpd, HSF targets) or slightly lower (0.5 cpd, LSF targets) spatial frequency (SF) than their respective standards.

Two attention conditions (attend-HSF and attend-LSF) were administered in random order in separate blocks of trials. Each block began with a 1000 ms presentation of a central fixation cross followed by the presentation of an attention-directing cue consisting of either the HSF or LSF standard grating. This cue instructed subjects as to which SF was task-relevant during the upcoming block of trials (i.e., an attend-HSF or attend-LSF block). The cue remained visible for 1000 ms and was followed by a variable interval (1200–2200 ms) during which only the fixation point was present on the screen. Following this interval, standard and target gratings were presented one at a time in random order at the center of gaze for 100 ms durations and stimulus onset asynchronies (SOAs) varying randomly between 700 and 900 ms. In each block, HSF and LSF stimuli were presented equiprobably and in random order. During the entire block of trials, subjects were instructed to maintain eye fixation on the central point (which was visible on the screen at all times) and respond with a button press to the target gratings, which deviated slightly from the attended standard SF. Each subject took part in 120 blocks of trials (60 attend-HSF blocks and 60 attend-LSF blocks), resulting in a total of approximately 336 presentations of both HSF and LSF standards when attended and unattended.

Correct detection rates (hits) and mean reaction time (RTs) to targets, and false alarms (FAs) to standards were recorded for each subject during the ERP experiment. Responses made within a window of 200–900 ms following a target or standard grating were considered hits and FAs, respectively. Separate repeated measures analyses of variance (ANOVA) were performed for each of these behavioral measures with a within-subject factor of SF (high or low) and a between subject factor of Group (Sz, control). Behavioral data were not available from two control and two patient subjects.

### Electrophysiological Recordings and Data Analysis

The ongoing electroencephalogram (EEG) was recorded using a custom Waveguard cap (Advanced Neuro Technology, ANT, Enschede, Netherlands) containing 64 equally spaced electrodes covering the whole head from slightly above the eyebrows to below the inion (Woldorff et al., 2002). Because of this special distribution of electrodes, only some electrode-positions completely match the International 10-20/10-10 system locations, in cases where they don’t match exactly the best estimate closest 10-20/10-10 location is given (a prime means it is within 0.5 mm of the listed 10-20/10-10 location; the suffix (a, i, m) signifies the site is 0.5–1.5 mm from the listed standard location in the direction given by the suffix (“a” for anterior, “i” for inferior, “m” for medial). Each channel was referenced to the average of all channels during recording. Impedances of all electrodes were kept below 5 kΩ throughout the experiment. Eye movements were monitored with bipolar electrodes placed on the left and right outer canthi to record the horizontal EOG.

Data were acquired at a sampling rate of 512 Hz and filtered offline using half-amplitude cutoffs of 0.1 and 100 Hz. ERPs from each electrode site were averaged separately for standard stimuli of each SF as a function of whether they were attended or unattended. In all cases, the averaged ERPs were digitally low-pass filtered with a Gaussian finite impulse function (3 dB attenuation at 46 Hz) to remove high-frequency noise produced by muscle activity and external electrical sources. Epochs with amplitudes exceeding ± 70 μV at any electrode were excluded from averaging. Additionally, ERPs elicited by standard stimuli that were preceded by a target stimulus within 900 ms were not included in the average. On average, 21% of the trials for patients and 14% of the control subjects’ trials were rejected.

As in previous studies, mean amplitude measures for the major sensory-evoked ERP components elicited by standard stimuli were taken within specified time windows encompassing the peak of each component. In all cases, a single measurement of mean amplitude was taken across each component window relative to the mean amplitude of the 100 ms baseline preceding stimulus onset. The C1 component elicited by HSF standards was measured in the latency window of 80–100 ms post-stimulus onset and the P1 component elicited by LSF standards was measured in the interval 100–120 ms. The mean amplitude of the C1 component was averaged across 3 midline electrodes (Inz, Oz’, POz) at which the component was maximal. Similarly, P1 amplitude was averaged across 12 electrode sites spanning the posterior scalp (6 in each hemisphere: PO1/PO2, O1’/O2’, O1i/O2i, I1/I2, TO1/TO2, TI1/TI2). Separate ANOVAs were performed for each component (C1 to HSF and P1to LSF standards) with a within-group factor of attention (attended, unattended) and a between-group factor of group (Sz, control).

Attention-related modulation of the longer-latency SN component was evaluated by comparing SN amplitudes elicited by attended standard stimuli of each SF with the SN elicited by the same stimuli when unattended. Between-group differences in these attend minus unattend SN amplitude differences for high and low SF were tested in separate one-way ANOVAs in the latency interval 200–400 ms post stimulus onset across the cluster of all 15 posterior electrode sites described earlier. The SN difference potential, formed by subtracting unattended ERPs from attended ERPs, was also calculated for individual subjects and its mean amplitude over the 200–400 ms interval was used in subsequent analyses.

### Time Frequency Analysis

To analyze oscillatory cortical activity associated with HSF and LSF gratings when attended and unattended, the single trial EEG signal on each channel was convolved with a five-cycle Morlet wavelet computed over a 3 s window centered at the onset of each stimulus. Instantaneous power and phase were extracted at each time point (at 512 Hz sampling rate) over 73 frequency scales from 0.73 to 53.5 Hz incremented logarithmically (Lakatos et al., 2005). Power was calculated as the sum of the squares of the real and imaginary Morlet components. The square roots of the power values, termed spectral amplitudes (in μV), were then averaged over the single trials separately for each stimulus type and electrode site to yield the total averaged spectral amplitude for each stimulus type and electrode site. The averaged spectral amplitude at each time point was baseline corrected by subtracting the mean spectral amplitude over the −150 to −50 pre-stimulus interval (corrected separately for each frequency band and in each individual subject; Tallon-Baudry et al., 1998). Additionally, the phase locking index (PLI) across trials was calculated by normalizing the complex wavelet decomposition on each trial by its absolute value and then averaging over all trials (Lakatos et al., 2005; Martínez-Montes et al., 2008). The PLI varies between zero and one and provides a measure of instantaneous spectral phase consistency at each frequency across all trials.

For statistical analyses, mean power was calculated within two latency intervals following stimulus onset, a 200–300 ms “early” interval and 500–700 ms; “late” interval. The ERD of the alpha (7–14 Hz) frequency band was measured as the reduction in alpha power from pre- to post-stimulus. Repeated measures ANOVAs were carried out on spectral power in each interval with factors SF (HSF, LSF), Attention (attended, unattended) and Group (controls, Sz patients). Similarly, repeated measures ANOVA were carried out on the sensory-evoked (i.e., time locked) activity in the 100–300 ms latency interval following stimulus onset and were based on PLI values for both LSF and HSF stimuli as a function of Attention and Group. In all cases, these statistical analyses were conducted over the same cluster of 15 posterior electrode sites that were used to test the ERP components.

### Symptom and Neuropsychological Measures

Symptoms of patients were assessed using the Positive and Negative Symptom Scale (PANSS; PANSS Institute, New York, NY, USA). General neuropsychological functions were assessed using the Measurement and Treatment Research to Improve Cognition in Schizophrenia (MATRICS) consensus cognitive battery (MCCB; Nuechterlein et al., 2008). This battery incorporates seven domains, including the Identical Pairs version of the Continuous Performance Test (CPT-IP), which is used to assess the domain of Attention/Vigilance, and the Brief Visual Memory Task, which is used to assess Visual Learning. The MCCB was available for 15 patients and 14 controls.

### Correlational Analyses

The interrelationship between behavioral performance, neurocognitive measures and electrophysiological indices of visual processing and selective attention, were evaluated in a sequence of hierarchical multiple linear regressions. Separate analyses were performed for the SN potential, alpha ERD during the early and late latencies, target discrimination accuracy (d’) and performance in the MATRICS attention-vigilance domain. In each set of regressions, group status (patient/control) and stimulus type (low/high SF) were entered at step 1, and additional measures were entered at step 2.

## Results

### Behavioral Performance

Behavioral results are shown in Figure 1B. Sz patients missed significantly more targets, particularly those of LSF stimuli, leading to a significant effect of group (*F*_(1,64)_ = 7.61, *p* = 0.008) and a group × SF interaction (*F*_(1,64)_ = 4.98, *p* = 0.029) for hit rate (Figure 1B, top). Similarly, patients showed significantly higher rates of FA to non-targets (i.e., standards) for both the LSF and HSF targets but with greater deficits during attention to LSF stimuli, leading to both a main effect of group (*F*_(1,36)_ = 10.60, *p* = 0.003) and a group × SF interaction (*F*_(1,36)_ = 6.22, *p* = 0.021; Figure 1B, middle). As a result, signal-detection analyses revealed significant deficits in d’ for both HSF (*F*_(1,17)_ = 7.59, *p* = 0.01) and LSF (*F*_(1,17)_ = 22.0, *p* < 0.0001) stimuli along with a significant group × SF interaction (*F*_(1,36)_ = 8.62, *p* = 0.007; Figure 1B, bottom). In contrast, the mean RT difference between patients and controls only approached significance (*F*_(1,36)_ = 3.34, *p* = 0.079) and did not differ between HSF or LSF targets (*F*_(1,36)_ = 1.53, *p* = 0.227).

### Time Domain Analyses (ERP)

#### Sensory-Evoked ERPs

**Figure 2:**
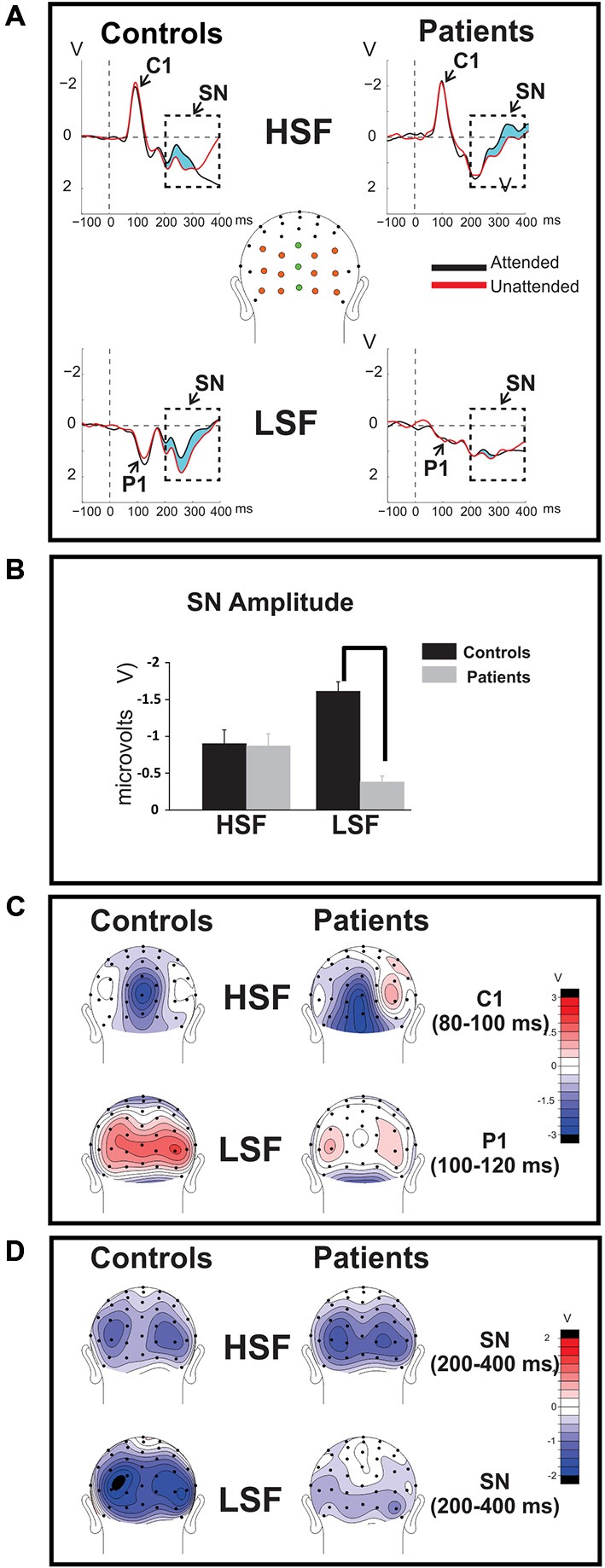
**Time-domain ERPs. (A)** Group-averaged ERPs elicited by attended (black tracings) and unattended (red tracings) standard stimuli of high (HSF; top row) and low (LSF; bottom row) SF are shown for Sz patients (right) and control subjects (left). The C1 component elicited by HSF was measured across midline electrode sites marked in green, the P1 component associated with LSF stimuli was measured across the electrodes marked orange. The attention-related modulation of stimuli of the attended SF, indicated by the light blue shaded area between the waveforms, was quantified as the difference between ERP amplitudes elicited by attended stimuli minus ERP amplitudes elicited by the same stimuli when unattended. The amplitude of the resulting selection negativity (SN) is shown in **(B)** for HSF (left bars) and LSF (right bars) standards for control subjects (blue bars) and Sz patients (red bars) in the bar graphs below. In all cases the SN was measured across the interval 200–400 ms post-stimulus onset over all colored electrodes shown in **(A)**. The SN to HSF stimuli was equivalent for control subjects compared to Sz patients but the SN was significantly diminished in patients for LSF stimuli. Error bars are standard error of the mean. (statistical significance is indicated by asterisks). **(C)** (top row): Mean scalp voltage topography of the sensory-evoked C1 component elicited by unattended HSF stimuli over the 80–100 ms latency interval following stimulus onset. (bottom row): Mean voltage topography of the P1 component (measured across the 100–120 ms latency window) elicited by unattended LSF. In contrast to the C1 component, which was relatively unimpaired in patients (right column) compared to controls (left column), the amplitude of the P1 was significantly reduced in patients with Sz. **(D)** The mean topographical distribution of the SN is shown for HSF (top) and LSF (bottom) standards across the interval 200–400ms post-stimulus onset.

Sensory-evoked ERPs to all standard stimuli were analyzed as a function of both SF and attended/unattended status. As in several previous studies, (e.g., Martínez et al., 2001; Baas et al., 2002), approximately 50 ms following stimulus onset an early negative deflection (the C1 component) was elicited by both attended and unattended HSF standards. C1 amplitude was not affected by attention (*F*_(1,36)_ = 1.54, *p* = 0.22) and was similar between Sz patients and controls (*F*_(1,36)_ = 1.55, *p* = 0.22; Figures 2A,C, top; Table 2, top). In contrast to HSF stimuli, LSF standards elicited an initial bilateral positivity over the dorsal occipital scalp (Figures 2A,C, bottom) with maximal amplitude in the interval 100–120 ms (the P1 component). As with the C1, the amplitude of the P1 was not modulated by attention in either Sz patients or control subjects [main effect of Attention: (*F*_(1,36)_ = 2.59, *p* = 0.12); Attention × Group: (*F*_(1,36)_ = 0.14, *p* = 0.71)]. However, the P1 amplitude to LSF stimuli was significantly lower overall for patients compared to control subjects across both attention conditions (*F*_(1,36)_ = 9.52, *p* = 0.003), suggesting an impaired initial cortical response to LSF, but not HSF stimuli, consistent with our prior findings (Martínez et al., 2012).

**Table 2:** **ERP amplitudes and PLI values**.

ERP Component		Mean (μV)	
		Controls	Patients	*t*_(36)_	*p*
C1 (HSF)	Attd.	−1.71	−1.92	1.48	0.16
(80–100 ms)	Unatt.	−1.89	−2.01	0.90	0.40
P1 (LSF)	Attd.	1.62	0.59	2.70	0.01
(100–120 ms)	Unatt.	1.44	0.30	3.01	0.00
**Theta (4–7 Hz) 100–300 ms**		**Mean PLI**	
		**Controls**	**Patients**	***t*_(36)_**	***p***
HSF	Attd.	0.25	0.22	1.01	0.32
Unatt.	Unatt.	0.29	0.22	1.46	0.15
LSF	Attd.	0.27	0.13	4.59	0.00
Unatt.	Unatt.	0.29	0.13	5.35	0.00

*(Top): Mean amplitudes of the C1 and P1 components (in microvolts, μV) elicited by attended (Attd.) and unattended (Unatt.) stimuli are given separately for control subjects (left column) and Sz patients (right column). The C1 was elicited by high (HSF) spatial frequency stimuli and the P1 component by low (LSF) frequency stimuli. Measurement latency windows, post-stimulus onset, are indicated for each component. *t*-values and significance (p) levels resulting from testing group differences (controls vs. patients) are given in two rightmost columns. Conditions with significant group differences are bolded. (Bottom): Mean phase locking index (PLI) measures (relative to pre-stimulus baseline) for HSF and LSF when attended (Attd.) and unattended (Unatt.) are given for control subjects and patients with Sz. Values were measured in the 100–300 ms latency range within the theta (4–7 Hz) frequency band. Associated t-tests and significance levels (p) are for the contrast of controls vs. patients. Conditions with significant group differences are bolded*.

#### Attention-Related ERP Effects

The SN was measured as the differential ERP elicited by standard stimuli when attended vs. unattended. A prominent SN was observed to both HSF and LSF stimuli in control subjects, consisting of a broad negative-going bilateral deflection over the ventral occipital scalp during the 200–400 ms latency range (Figure 2A; shaded region, Figures 2B,D), consistent with previous studies of selective attention to SF (e.g., Zani and Proverbio, 1995; Martínez et al., 2001; Baas et al., 2002). As in our prior study (Martínez et al., 2012), patients and controls showed similar SN amplitudes to HSF stimuli but patient amplitudes were reduced to LSF stimuli.

### Time-Frequency Analyses

**Figure 3:**
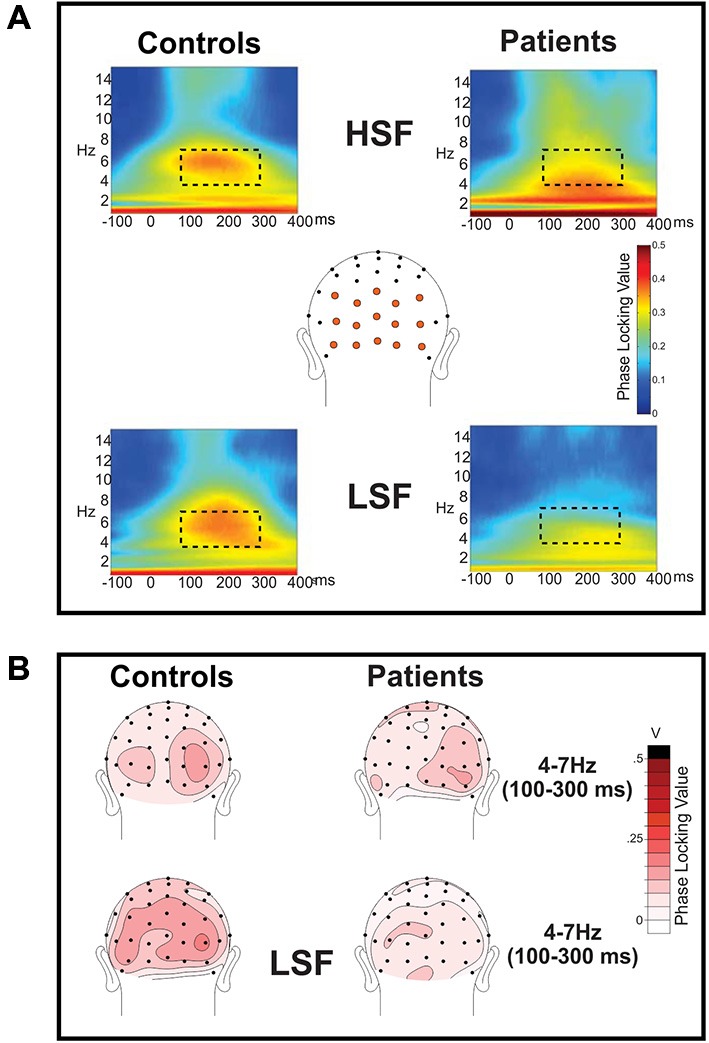
**Frequency domain analysis of event-related spectral perturbations: phase locking index (PLI). (A)** Phase locking index (PLI) values were calculated for the spectral perturbations to high (HSF; top row) and low (LSF; bottom row) SF standard stimuli for controls (left) and patients with Sz (right). PLI values were measured across the electrode sites shown in orange and are shown collapsed across attended and unattended conditions, which did not significantly differ. For both Sz patients and controls, there was clear phase locking in the theta frequency band (4–7 Hz) which was significantly reduced in patients for LSF, but not HSF, stimuli. The dotted rectangle indicates the time (100–300 ms)—frequency (4–7 Hz) window used for statistical analysis. **(B)** Scalp topography of PLI values for HSF (top) and LSF (bottom) stimuli in the 100–300 ms latency interval. Occipital topographies of PLI values elicited by LSF stimuli show diminished amplitudes in Sz patients (right) vs. healthy control subjects (left) whereas PLI topographies for HSF stimuli were similar for the two groups.

As reported previously (Mishra et al., 2012; Dias et al., 2013), the initial sensory response was reflected in increased stimulus-evoked (phase-locked) theta band (4–7 Hz) activity over the occipital scalp that encompassed the P1/C1 latency interval (100–300 ms; Figure 3). In particular, Sz patients showed highly significant reductions in theta phase synchrony (PLI) to LSF (*F*_(1,36)_ = 23.2, *p* < 0.0001) but not HSF (*F*_(1,36)_ = 1.97, *p* = 0.17) stimuli relative to controls within this 100–300 ms interval leading to a significant main effect of group across SF (*F*_(1,72)_ = 6.91, *p* < 0.0001) as well as a significant group × SF interaction (*F*_(1,72)_ = 6.91, *p* = 0.01). By contrast, neither the main effect of attention (*F*_(1,72)_ = 1.48, *p* = 0.24), the group × attention interaction (*F*_(1,72)_ = 1.51, *p* = 0.22) nor the SF × attention interaction (*F*_(1,72)_ = 0.08, *p* = 0.78) was significant for PLI values (Table 2, bottom).

**Figure 4:**
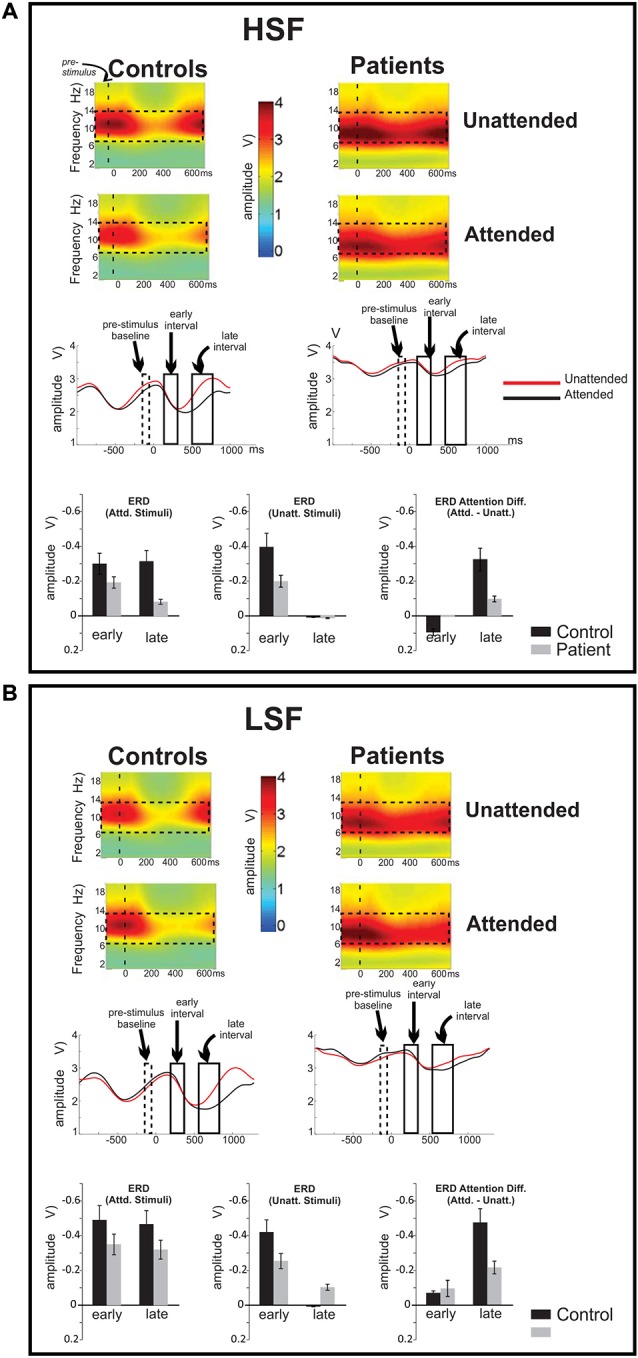
**Frequency domain analysis of event-related spectral perturbations**. Ongoing EEG activity and stimulus related perturbations are shown for **(A)** high (HSF) and **(B)** low (LSF) SF standards for control subjects (left column) and Sz patients (right column), averaged across the cluster of 15 electrode sites denoted in Figure 3A. In both subject groups, ongoing EEG activity was observed primarily in the broad alpha (7–14 Hz) frequency band (denoted by dashed rectangles on the spectral plots). Compared to control subjects, alpha amplitude was greater overall in patients with Sz. Stimulus presentations occurred at time 0 (vertical dashed line on spectral plots). Onset of both HSF and LSF stimuli elicited a significant reduction of ongoing alpha-band activity, which was prolonged for attended relative to unattended stimuli. Across attention conditions, this reduction in ongoing alpha activity was significantly less in Sz patients compared to control subjects. The time course of these modulations of alpha activity are shown on waveforms for attended (red tracings) and unattended (red) stimuli of **(A)** HSF and **(B)** LSF standards.

The power of ongoing spectral perturbations were centered in the alpha frequency band (7–14 Hz) for both Sz and control subjects. To evaluate task-related amplitude modulations of this ongoing alpha, mean spectral amplitudes within the alpha band were averaged over 2 s intervals centered on presentations of standard stimuli when attended and unattended (Figures 4A,B, top). In this frequency band, oscillatory power (measured during the −150 to −50 ms pre-stimulus/baseline latency window) was significantly higher amplitude in Sz patients compared to healthy control subjects (*F*_(1,36)_ = 15.27, *p* < 0.001).

In response to the onsets of standard stimuli (either high or low SF), a large amplitude reduction (event-related desynchronization, ERD) in alpha activity was observed beginning approximately 100 ms following stimulus onset This stimulus induced modulation was analyzed by baseline-correcting the alpha amplitude to the pre-stimulus period. The ERD reached its maximum at approximately 500 ms following unattended stimuli but was prolonged following attended stimuli, leading to an amplitude differential as a function of attention during the 500–700 ms latency range. Accordingly, statistical analyses were conducted within two latency intervals, an early interval (200–300 ms) corresponding to the initial stimulus driven ERD and a late interval (500–700 ms) where the effects of attention were maximal (Figures 4A,B, middle, bottom).

When analyses were conducted across the early and late ERD intervals, the effect of group was highly significant (*F*_(1,72)_ = 16.4, *p* = 0.0001), with no significant group × latency interval (*F*_(1,72)_ = 1.29, *p* = 0.3) or group × SF (*F*_(1,72)_ = 0.03, *p* = 0.9) interaction. Further statistical analyses of these values showed significant differences between the groups in alpha reductions (ERD) in both the early (*F*_(1,36)_ = 7.07, *p* = 0.011) and late (*F*_(1,36)_ = 5.50, *p* = 0.025) latency intervals with healthy controls showing a greater ERD overall than Sz patients in both cases.

When analyzed separately, ERDs in the early interval were marginally larger in controls than in patients (*F*_(1,36)_ = 4.08, *p* = 0.051) but were similar to HSF and LSF stimuli, as reflected in a non-significant main effect of SF (*F*_(1,36)_ = 0.0003, *p* = 0.986). Further, in this interval there was no significant difference between the ERD elicited by attended and unattended stimuli in either group (*F*_(1,36)_ = 1.11, *p* = 0.297). By contrast, in the late interval, the effect of attention was robust in both subject groups, with larger ERD amplitudes for attended compared to unattended standard stimuli (*F*_(1,36)_ = 34.98, *p* < 0.001). This effect of attention was greater for LSF compared to HSF stimuli as evidenced in the attention × SF interaction (*F*_(1,36)_ = 18.83, *p* = 0.006) over both groups with no attention × SF × group interaction (*F*_(1,36)_ = 2.10, *p* = 0.156). Finally, for both HSF and LSF, the effects of attention on alpha ERD amplitude in the late interval were significantly larger overall in control subjects compared to patients with Sz (*F*_(1,36)_ = 10.68, *p* = 0.002).

### Correlational Analyses

**Figure 5:**
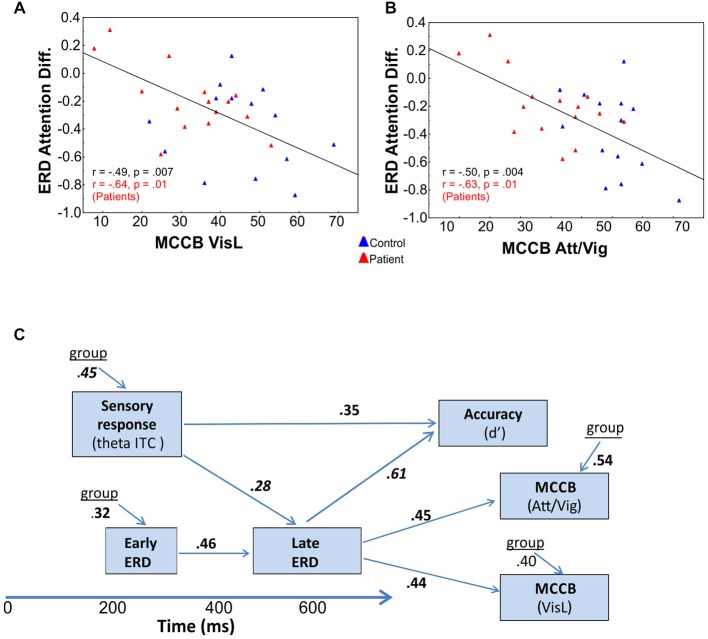
**Correlational analyses**. Correlation of late attention-related modulation of event-related desynchronizations (ERD) with **(A)** visual learning (VisL) and **(B)** attention/vigilance scales (Att/Vig) of the MATRICS consensus Cognitive Battery (MCCB). In both cases, improved scores were associated with larger amplitude ERD changes in the late interval as a function of attention, both across groups (values in black) and for Sz patients alone (values in red). **(C)** Interrelationship among oscillatory measures and behavior. Deficits in initial theta-band PLI and alpha ERD responses to visual stimuli significantly predicted impaired behavioral performance (d’) on the present task, as well as impaired performance on visual domains of the MCCB, including the attention/vigilance (Att/Vig) and visual learning (VisL) domains, over and above contributions of diagnostic group. These correlations were also independently significant within the patient group alone.

Stepwise regression analyses were conducted to investigate the interrelationship among time- and frequency-domain events as well as between event-related neural activity and higher-order neurocognitive measures. Across SF, the amplitude of the initial phase locked theta PLI response to unattended stimuli was strongly correlated with group membership (*F*_(2,73)_ = 9.63, *p* < 0.0001; partial *r* = 0.45, *p* < 0.0001). The amplitude of the (unattended) alpha ERD in the early interval was also significantly correlated with group membership (*F*_(2,73)_ = 9.61, *p* = 0.002; partial *r* = 0.32, *p* = 0.005). Additionally, the magnitude of this early alpha ERD was significantly associated with the magnitude of the attention-related modulation of alpha ERD in the late interval (*F*_(3,72)_ = 6.04, *p* < 0.0001, partial *r* = 0.46, *p* = 0.001) as was the magnitude of the initial theta-band PLI response (*F*_(3,72)_ = 6.37, *p* < 0.0001; partial *r* = 0.28, *p* = 0.016; Figure 5). Finally, the amplitude of the theta PLI response at 100–300 ms was significantly correlated with the amplitude of the subsequent SN ERP potential (200–400 ms) across groups and across stimulus types (*F*_(3,72)_ = 10.38, *p* < 0.0001; partial *r* = −0.34, *p* = 0.003).

Electrophysiological responses were additionally analyzed relative to behavioral performance. Potential predictors in the hierarchical regression model (in addition to group and SF), included the initial theta-band response, the late attentional ERD and the SN potential. Both the initial theta-band PLI response (*F*_(3,54)_ = 6.91, *p* < 0.0001, partial *r* = 0.35, *p* = 0.005) and the late alpha ERD attention effect (*F*_(3,54)_ = 14.52, *r* = 0.61, *p* < 0.0001) were significant independent predictors of target discrimination accuracy (d’). The overall model incorporating these measures was highly significant (adj. *R*^2^ = 0.72, *F*_(4,53)_ = 14.6, *p* < 0.0001). When these oscillatory values were entered into the regression analyses, group accounted for only an additional 1.8% of the variance. The amplitude of the SN, when considered individually was also a significant predictor of target discrimination accuracy (*F*_(1,54)_ = 8.7, *p* = 0.004, partial *r* = 0.35, *p* = 0.003) over and above the contributions of group.

Cognitive data, as measured by the MATRICS consensus cognitive battery (MCCB), were available for 15 patients and 14 controls. A final analysis evaluated predictors of performance on the MATRICS Attention/vigilance and Visual Learning domains and oscillatory measures of visual processing (the initial theta PLI response and the late and early alpha ERD were considered as potential predictors). First, patients showed highly significant deficits across all MCCB domains (Table 3). When stepwise regression analyses were applied to these data, the amplitude of the late alpha ERD to LSF stimuli significantly predicted performance on the Attention-vigilance (adj. *R*^2^ = 0.55, *F*_(4,18)_ = 5.57, *p* = partial, *r* = 0.45, *p* = 0.041) and Visual learning (adj. *R*^2^ = 0.38, *F*_(4,18)_ = 4.06, *p* = 0.018, partial *r* = 0.44, *p* = 0.019) domains over and above the highly significant effects of group. These correlations remained significant within the Sz group independently (Att/vig: *r* = 0.54, *p* = 0.031; VisL: *r* = 0.64, *p* = 009). Correlations with other MCCB domains were non-significant. As compared to neuropsychological test data, no significant correlations were observed between oscillatory activity and medication dose (CPZ equivalents).

**Table 3:** **MATRICS domain scores**.

MATRICS Domain	Controls	Patients	*t*_(29)_	*p*
Speed of Processing	50.29	24.87	5.89	0.00
Attention/Vigilance	47.87	28.88	4.56	0.00
Working Memory	48.29	30.40	4.12	0.00
Verbal Learning	44.14	31.93	4.36	0.00
Visual Learning	45.43	32.47	2.76	0.01
Reasoning/Problem Solving	45.14	33.00	2.81	0.01

*Neurocognitive composite scores from six domains in the MATRICS Consensus Cognitive Battery for normal control subjects and patients with Sz. Associated t-scores and significance levels (p) are given for the comparison of controls vs. patient scores on each domain. In all cases patients performed significantly worse than controls*.

## Discussion

Deficits in early visual processing in Sz are extensively documented as reflected in behavioral (Cadenhead et al., 1998; Chen et al., 2000; Brenner et al., 2002; Li, 2002), fMRI (Martínez et al., 2008, 2013) and time-domain ERP results (Ford et al., 2001; Foxe et al., 2001; Butler et al., 2007; Dias et al., 2011; Martínez et al., 2012; González-Hernández et al., 2014). These deficits are associated with impairments in higher level processes such as emotion recognition (Gur et al., 2002; Butler et al., 2009), feature selection (Alain et al., 2002; Mishra et al., 2012) and reading (Revheim et al., 2006; Martínez et al., 2013). In the present study, we examined both time and frequency domain neural activity that may underlie impaired visual processing in Sz during a feature-selection task known to be sensitive to magnocellular dysfunction (Martínez et al., 2012). These physiological indices of visual processing impairment were correlated with behavioral and neurocognitive measures to examine their contribution to higher order cortical deficits in Sz.

In addition to confirming our earlier reports of impaired generation of time-domain ERP components such as the P1 and the SN to magnocellularly-biased (LSF), stimuli, we now demonstrate significant SF-independent impairment in the modulation of ongoing alpha-band activity in Sz that correlates with behavioral and neuropsychological deficits in visual information processing. These findings suggest an additional mechanism by which deficits in early visual processing of both HSF and LSF stimuli contribute to overall cognitive impairment in Sz. In addition, these results provide the first evidence that stimulus-induced alpha modulation can be prolonged in a top-down fashion by feature-selective attention. The relative SF-dependence of ERP generation compared to the SF-independence of alpha modulation, moreover, provides key information regarding potential underlying neural substrates.

Consistent with our previous report (Martínez et al., 2012), patients in the current study showed differential deficits relative to controls when asked to attend to LSF rather than HSF stimuli. In the time domain, patients with Sz showed significant amplitude reductions in both the P1 and SN components elicited by LSF stimuli, with relatively intact sensory-evoked and attention-related ERPs to HSF stimuli. These ERP deficits were echoed in a virtually absent theta-band phase locked response to both unattended and attended LSF stimuli, supporting our prior reports of impaired sensory transmission within the early magnocellular system (Martínez et al., 2012).

In contrast to the theta-band evoked responses that were tightly stimulus locked and reflected therefore in PLI analyses, stimulus-induced desynchronizations of ongoing alpha activity were not tightly stimulus locked in either group and were therefore apparent only in induced power analyses. Moreover, in contrast to theta phase locked responses, which were differentially impaired to LSF stimuli in patients, the early alpha ERD was equivalently reduced to both LSF and HSF stimuli in Sz patients; that is, similar levels of impairment in alpha ERD generation were observed for HSF and LSF responses. These findings suggest that stimulus-induced alpha modulation represents a separate domain of neurophysiological dysfunction from previously described impairments in sensory ERP generation in schizophrenia, with complementary contribution to visual information processing. In support of this proposal, target detectability measures (d’) were found to be highly correlated with the attention-related modulations of alpha rhythm.

In addition to an inability to modulate ongoing alpha activity during visual processing, we found that ongoing alpha power measured during the baseline (prestimulus) interval was significantly elevated in Sz patients compared to controls. Increased ongoing oscillatory activity has been reported previously in patients with Sz both in the alpha and theta frequency bands (Hanslmayr et al., 2013; Lakatos et al., 2013). The functional and clinical significance of these abnormally high brain oscillations remains unknown, however, it has been associated with a failure to increase oscillatory activity during cognitive tasks (Hanslmayr et al., 2013).

An additional new finding in the present study is that, while the magnitude of the initial alpha ERD was attention-independent in both Sz patients and controls, the duration of the ERD was significantly modulated by feature attention. Specifically, for unattended stimuli the initial ERD began to resolve by approximately 300 ms, independently of SF. By contrast, for attended stimuli, peak ERD levels persisted until approximately 500 ms, and the ERD did not fully resolve until about 800 ms. We hypothesize that the prolonged ERD was associated with additional time for stimulus evaluation and response selection processes following the attended stimulus. ERD responses in this late interval to attended stimuli were similar in magnitude and strongly correlated with the amplitude of the same stimuli in the early interval, suggesting that the late phase reflects an attention-dependent prolongation of a stimulus-induced ERD rather than a de novo attention-induced effect.

Quantification of the attention effect on alpha ERD prolongation was operationalized in the present study by measurement of the difference in late ERD response to attended vs. unattended stimuli. In Sz patients, reduction in the alpha ERD attention effect correlated highly with impaired behavioral performance not only on the feature selective attention task itself but also on visually based neuropsychological tasks within the MATRICS consensus cognitive battery for schizophrenia, including scores on the Attention/vigilance and Visual learning domains. Impaired function on these tasks, in turn, is known to correlate with impaired daily functioning (Kern et al., 2011; August et al., 2012). The fact that patients’ deficits did not correlate with antipsychotic dose suggests that the observed impairments cannot be attributed to effects of antipsychotic medication.

Both the early PLI response and the early alpha ERD were independently correlated with the late attention-related modulation of alpha activity which, in turn, was itself predictive of behavioral response accuracy and neuropsychiatric clinical measures. This suggests that critical pathological processes in SZ involve the initial stimulus-evoked response and stimulus-induced modulation of ongoing alpha activity, which are predictive of the subsequent attention-dependent alpha modulation.

It has been proposed that suppression of ongoing alpha activity is essential for bringing brain regions “on line” so that they can engage in appropriate task-dependent processing (Yamagishi et al., 2003; Rihs et al., 2007; Klimesch, 2012). The present study thus suggests that failures in occipital alpha modulation may lead to significant impairments in visual information processing in Sz. Our present findings of reduced magnitude of alpha ERD in Sz correspond well to reports of impaired “alpha blocking” in Sz dating back to the 1930’s (MacMahon and Walter, 1938; Salamon and Post, 1965). Nevertheless, this is the first study to relate this reduced alpha blocking to deficits in visual information processing and attention/vigilance in Sz.

We are aware of only one other study that investigated the integrity of stimulus-driven alpha activity in schizophrenia. In that study, the phase locking of alpha-frequency steady-state visual evoked potentials (SSVEPs) was used as the measure of early visual activity (Clementz et al., 2008). Because no deficits were observed in alpha phase locking it was concluded that sensory processing was intact and only subsequent stages of processing were impaired in Sz. The present study demonstrates highly significant impairments in alpha ERD in schizophrenia that can be detected by measuring alterations in single-trial power of ongoing oscillatory activity rather than phase locking of responses across trials and illustrates the need for assessment of oscillatory power, as well as phase locking, in pathological studies.

Over recent years, alpha generation has been linked to both muscarinic and mGluR1a-mediated glutamatergic transmission within thalamus (Hughes and Crunelli, 2005). The deficits observed in the present study are thus consistent with prevailing cholinergic (Olincy and Freedman, 2012), GABAergic (Gonzalez-Burgos and Lewis, 2012) and glutamatergic (Javitt, 2010) models of Sz. However, in addition to providing insights into the mechanisms underlying visual processing impairments in Sz, the present study may also give insights into neurophysiological mechanisms underlying normal alpha-band modulation.

In particular, whereas LSF and HSF stimuli elicited markedly different initial sensory responses in the time-domain, consistent with preferential engagement of magnocellular vs. parvocellular pathways (Livingstone and Hubel, 1987; Tootell et al., 1988), alpha ERD was similar in response to LSF and HSF stimuli suggesting that the neural mechanisms giving rise to the initial alpha ERD may not rely on traditional magnocellular and parvocellular pathways. The neurophysiological substrates of the attention-dependent prolongation of the alpha ERD are unknown. Nevertheless, prominent alpha synchrony has been reported between pulvinar nucleus and visual cortex regions during visual attention (Saalmann et al., 2012), suggesting a potential role of pulvinar in this process. In addition to theta and alpha abnormalities, deficits in delta band ITC have also been reported during auditory (Lakatos et al., 2013) and visual (Ergen et al., 2008) processing in schizophrenia. Although the present study did not analyze delta range activity, future studies assessing delta activity would be warranted.

In summary, the present study demonstrates that Sz patients show significant decrements in alpha blocking during cognitive task performance and that these deficits correlate strongly not only with impaired performance in the feature-attention task itself, but also in attention/vigilance and visual learning tests frequently used to assess higher level cognitive dysfunction in schizophrenia. Overall, these findings suggest that impaired ability to bring appropriate brain regions “on line” through alpha desynchronization may contribute substantially to impaired cognitive dysfunction in Sz.

## Conflict of Interest Statement

The authors declare that the research was conducted in the absence of any commercial or financial relationships that could be construed as a potential conflict of interest.

## Footnotes

^1^http://www.neurobs.com/
